# Towards ultrahigh resolution OCT based endoscopical pituitary gland and adenoma screening: a performance parameter evaluation

**DOI:** 10.1364/BOE.409987

**Published:** 2020-11-09

**Authors:** Fabian Placzek, Alexander Micko, Ryan Sentosa, Roger Fonollà, Michael Winklehner, Arthur Hosmann, Marco Andreana, Romana Höftberger, Wolfgang Drexler, Rainer A. Leitgeb, Stefan Wolfsberger, Angelika Unterhuber

**Affiliations:** 1 Center for Medical Physics and Biomedical Engineering, Medical University of Vienna, Waehringer Guertel 18-20, 4L, 1090 Vienna, Austria; 2 Department of Neurosurgery, Medical University of Vienna, Waehringer Guertel 18-20, 1090 Vienna, Austria; 3 Department of Electrical Engineering, Video Coding and Architectures, Eindhoven University of Technology, 5612 AZ Eindhoven, Noord-Brabant, The Netherlands; 4 Division of Neuropathology and Neurochemistry, Department of Neurology, Medical University of Vienna, Waehringer Guertel 18-20, 1090 Vienna, Austria; 5 Christian Doppler Laboratory OPTRAMED, Medical University of Vienna, Waehringer Guertel 18-20, 1090 Vienna, Austria; 6 These authors contributed equally to this work

## Abstract

Ultrahigh resolution optical coherence tomography (UHR-OCT) for differentiating pituitary gland versus adenoma tissue has been investigated for the first time, indicating more than 80% accuracy. For biomarker identification, OCT images of paraffin embedded tissue are correlated to histopathological slices. The identified biomarkers are verified on fresh biopsies. Additionally, an approach, based on resolution modified UHR-OCT *ex vivo* data, investigating optical performance parameters for the realization in an *in vivo* endoscope is presented and evaluated. The identified morphological features–cell groups with reticulin framework–detectable with UHR-OCT showcase a promising differentiation ability, encouraging endoscopic OCT probe development for *in vivo* application.

## 1. Introduction

Pituitary adenomas are intracranial tumors, appearing in 15% of affected patients [1]. Standard surgical procedure is resection of the adenoma through transnasal transsphenoidal surgery [2,3]. The goal of this approach is the complete removal of the adenoma under the visualization with rigid endoscopes introduced through the nose, sphenoidal sinus cavity and entering the sella turcica, where the pituitary gland is located [4]. Besides, the gross total removal of the tumor, the preservation of the normal pituitary gland is crucial. Dependent on adenoma size, invasiveness and endocrinological state, the complete tumor resection is achieved in 40-92.3% [5]. The gross total resection is limited by the difficult discrimination of adenoma and adjacent pituitary gland due to textural similarities, which causes tumor remnants and impairment of pituitary function if gland tissue is inadvertently removed. Furthermore, incomplete adenoma resection may lead to persistent clinical symptoms, recurrent tumor growth and repeated surgery with potential medical and radiosurgical treatment [6]. Failure to preserve the function of the normal pituitary gland causes hormonal insufficiencies and may require lifelong medical treatment.

On a cellular level, the pituitary gland expresses glandular structures, leading to morphological differentiation capabilities of pituitary versus adenoma tissue. In case of gland tissue cell conglomerations, also called nests or acini [7], are present. The size of acini is varying within the pituitary gland [8]. Those nests are responsible for the hormonal expression and as such are extremely important for hormone driven processes within the human body. Furthermore, intact reticulin fibers, composed of collagen, are present in gland tissue as a fiber framework, confining the cell nests. In contrast, pituitary adenoma are characterized by complete disruption of the reticulin confinement [8] and the nest-like structure gets lost [9]. This biomarker is well-known for morphological differentiation of pituitary gland and adenoma tissue based on histopathological slices [10,11]. However, histopathological examination, requiring paraffin embedded tissue samples, is time consuming. Therefore, novel methods for intraoperative identification of pituitary adenoma tissue are in significant need.

Optical coherence tomography (OCT) as one of the most innovative and successfully translated medical imaging techniques across the medical disciplines may be a useful imaging modality to be employed for this purpose. It is a non-invasive method to access depth resolved cross-sectional information (B-scan) at micrometer resolution [12]. Therefore, OCT provides morphological structure used for identification of pathologies. This imaging technology is translated to ophthalmology [13], urology [14,15] or neurosurgery where OCT is able to detect glioma margins from white matter [16,17]. Recently, OCT has been increasingly transferred to endoscopic assessment of internal organs through miniaturized imaging systems, e.g. in the cardiovascular system, the urinary tract [18–20] or the brain [21]. In general, bright OCT signal is caused by the presence of collagen fiber rich tissue [22]. Moreover, in case of glandular tissue, OCT has been successfully applied to detect gastrointestinal adenoma tissue and to intraoperatively identify parathyroid tissue [23].

In this work, we evaluate the use of *ex vivo* microscope based ultrahigh resolution spectral domain OCT (UHR-SD-OCT) at 800 nm for differentiating pituitary gland from adenoma tissue and map the findings directly to histology. The isotropic resolution of the UHR-SD-OCT reveals optical performance comparable to histopathological images. It enables identification of subtle structural features for a differentiation of pituitary gland from adenoma tissue. Out of this correlation diagnostic biomarkers for *in vivo* endoscopic OCT imaging are established, which can serve as differentiation criteria in daily clinical practice. The verification of these criteria is performed on fresh biopsies. Axial and lateral resolution modified data sets are created and analyzed by a support vector machine (SVM) to investigate diagnostic classification capabilities. For discriminating adenoma and gland tissue accuracies higher than 80% are reached. This approach enables the assessment of design parameters for endoscopic probes to be used for *in vivo* differentiation of pituitary gland and adenoma intraoperatively.

## 2. Material and methods

### 2.1 Biopsy preparation

The study was approved by the ethics committee of the Medical University of Vienna (EK-number: 1286/2018). The biopsy was performed during standard transsphenoidal surgery. Two different types of samples were used: I) Freshly unfrozen biopsies, which were snap frozen and stored at -80°C directly after resection and II) Biopsy specimen embedded in paraffin.

**Fig. 1.**
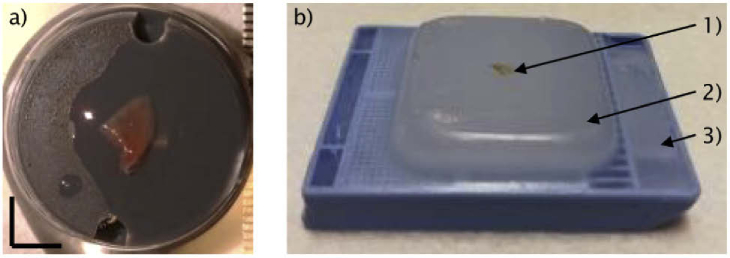
Biopsy preparation. a) The unfrozen biopsy is put onto custom made washers, placed inside the microscope holder and moisturized with saline solution. Scale bar: 3 mm. b) The fixed and embedded biopsy is displayed (1). The paraffin wax is holding the entire biopsy for subsequent slicing (2). Cassette, holding the paraffin block (3).

For the first type, a part of the resected adenoma is snap frozen for subsequent imaging. If adjacent gland tissue adherent to the pituitary adenoma was resected as a routine procedure for estimating invasive growth, these samples served as the control tissue samples. For OCT imaging, the snap frozen biopsies were thawed, placed on a microscope holder (D35-14-1.5N – Cellvis) and moisturized with saline solution (Fig. 1(a)). After OCT examination, all biopsy specimens were fixed in 4% neutral buffered formalin, routinely processed, embedded in paraffin, cut in 2 μm slices and stained with hematoxylin and eosin (H&E) for routine histopathological examination providing the ground truth labels. For the second type, already embedded biopsies were taken. In routinely performed histopathological work-up, those are fixed in 4% neutral buffered formalin, routinely processed and embedded in paraffin wax. For everyday usability, the paraffin block is fixated onto a cassette (Fig. 1(b)).

**Fig. 2.**
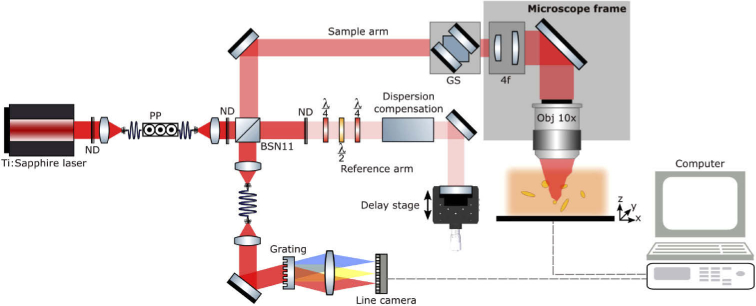
Optical setup, adapted from Andreana *et al*. [24]. The previously presented UHR-SD-OCT system is now in a free space Michelson interferometer configuration using a 10:90 BSN11 beam splitter from Thorlabs. 10% of the incident power is guided to the sample arm, ensuring 90% throughput of the collected signal to the spectrometer resulting in a measured sensitivity of 101 dB. Moreover, the imaging system is incorporated into an inverted microscope frame (Zeiss Observer Z1). ND: Neutral density filter, PP: polarization paddles, GS: galvanometric scanners, 4f: Telescope system for expanding the beam diameter, λ/4: quarter-wave plate, λ/2: half-wave plate.

The cassette was directly placed on the microscope frame of the optical setup (see Fig. 2) for OCT investigation of embedded tissue samples. The ground truth label was provided by the pathological report, performed prior to the OCT investigation.

In total, 20 freshly unfrozen biopsies were imaged. 10 samples were pituitary gland tissue and 10 biopsies were adenoma tissue. Each pair of gland and adenoma tissue originated from the same subject. Seven cassettes holding embedded tissue, with 4 containing gland and 3 adenoma tissue, were examined by OCT.

### 2.2 Optical coherence tomography

A modified version of a UHR-SD-OCT system described elsewhere was used in this study [24,25]. In brief, the system (Fig. 2), incorporated a compact Ti:Sapphire laser with a central wavelength at 800 nm and a bandwidth of 150 nm at the full width half maximum. The adapted free-space system was implemented into an inverted microscope (Zeiss Observer Z1) and achieved a measured sensitivity of 101 dB with sample incident power of 2.2 mW. The achievable sensitivity was limited due to an overfilling of the objective back aperture and the overall losses in the microscope frame. The light was focused by a 10x objective (Nikon CFI Achro 10× 0.25NA) resulting in a measured lateral resolution of 1.4 µm in air using the edge spread function (ESF) method [26]. The effective bandwidth of 96 nm resulted in a measured axial resolution of 2.9 µm in air, corresponding to a ∼2.2 µm axial resolution in tissue, assuming a refractive index of 1.36 for grey brain matter [27]. Galvanometric scanning mirrors performed a two-dimensional scanning over a field of view (FOV) of 0.9 × 0.9 mm^2^. The FOV was sampled with 1600 × 1600 points resulting in 2.5 times oversampling. The line camera was able to acquire A-scans with up to 70 kHz and each OCT data set was resolved by 1024 pixels in depth after Fourier transformation (FFT). The measured pixel size in depth after FFT was 1.3 µm, resulting in ∼1 µm in tissue. Thereby, the scanned volume had a size of 0.9 × 0.9 × 1 mm^3^. The UHR-SD-OCT system incorporated a motorized translation stage (*Physik Instrumente –* C-867) to choose specific region of interests (ROI) within the samples.

### 2.3 Correlation of morphological features

For morphological feature correlation in the OCT images with histology, the embedded biopsies were imaged first (Fig. 1(b)). The cassette, with the embedded biopsy, was placed directly onto the motorized translation stage and different ROIs could be selected from the real time preview. After the OCT data was acquired, the cassette was taken to standard pathological processing: slices with a thickness of ca. 2 µm were cut and stained with H&E. In between each slice there was material additionally cut which was not considered in the slicing procedure. The H&E slices were scanned with a digital microscope providing the capability of analyzing and comparing OCT and H&E images on a computer. The correlation procedure is described in more detail in the result section.

The relevant structural features are based on the cell nests confined by collagen fibers in gland tissue and the loss of this morphological architecture in the adenoma tissue. After those features were identified as detectable biomarkers for discrimination of healthy and pathological tissue, they were discussed and confirmed by histopathologists. As a next step, freshly unfrozen biopsies (Fig. 1(a)) were screened for the previously established biomarkers. The findings were discussed and verified by clinicians and histopathologists to ensure a correct translation to unprocessed tissue. The OCT images of the freshly, unfrozen biopsies are giving insight on expectable features, detectable during endoscopic assessment during surgery.

### 2.4 Image and texture analysis

The raw OCT data is processed with an in-house programmed MATLAB software. OCT data sets are visually analyzed and further processed with Fiji: representative B-scans are selected, a present lookup table termed “gold” is applied and brightness/contrast settings are adjusted. First, the OCT data of the embedded tissue and the corresponding H&E images are analyzed simultaneously to achieve a colocalization of the images. The surrounding signals outside the biopsy were removed from the enface images and B-scans using Fiji. Since the H&E slice represents similar features to an enface OCT image, the OCT data is resliced to be investigated from the enface direction. Out of the correlation of H&E and OCT enface images, morphological biomarkers are identified. In a next step, those biomarkers are assessed for freshly unfrozen biopsies. Representative samples for gland and adenoma tissue are presented with the same brightness and contrast settings to enable comparison of signal intensities. The signals outside of the representative enface signals of the pituitary gland were removed by using the software Fiji.

Texture analysis is a simple but yet effective, automated method in identifying patterns on OCT images [28,29]. Therefore, texture analysis was successfully applied in previous studies [30,31] and consequently a similar approach was chosen in this study: For each B-scan, the gray-level co-occurrence matrix (GLCM) was computed to extract textural spatial information. The energy, contrast, homogeneity and correlation were calculated at 0, 45, 90, and 135 degrees and used as main features for the automatic tissue differentiation. Prior to the feature extraction, each B-scan was preprocessed to ensure enough texture coverage and optimal feature selection: for each volume a total of 500 adjacent B-scans were selected as representatives concentrated in the middle of the biopsy. Each selected B-scan was cropped to a fixed 200 × 500 pixel region which included tissue and discarded surrounding noise and background signal. A total of two patches (200 × 200 pixels) were selected for each B-scan to compute the GLCM features, hence 1.000 patches with 16 features each were created for each biopsy. This resulted in 10.000 patches available for training, belonging to 10 biopsies: 5 normal pituitary glands and 5 adenomas. Biopsies were paired combining corresponding gland and adenoma tissue, taken from the same subject. The data was normalized before performing the feature analysis of the principal component analysis (PCA) and SVM.

### 2.5 Translation to endoscopic imaging performance

After the high imaging performance of the UHR-SD-OCT system, the potential differentiation possibilities of data that could be expected from *in vivo* endoscopic systems in the future were investigated. Due to the diameter constraint of endoscopes, they exhibit in general lower numerical apertures (NA) than microscopy systems resulting in lower lateral resolution. To mimic the optical performances of endoscopic probes, the axial and lateral resolution of the acquired microscopic OCT image data was reduced. Since axial and lateral resolution are decoupled in OCT, both were reduced as followed: The raw-spectra of the point wise depth scans (A-scans) were filtered with a Gaussian function. Knowing the full width at half maximum (FWHM) of these Gaussian window functions, the bandwidth was reduced. Using a mirror as a sample, the point spread function (PSF) was determined to get the adapted axial resolution. Axial resolutions in the range of 3-20 µm are achieved in various designs for OCT endoscopes and the applied light sources [20,26]. Therefore, the axial resolution was adapted to the following values: 5.8 µm, 11.5 µm, 17.3 µm.

Respectively, the lateral resolution was reduced with the following method. First, the bulk motion, mainly induced through an additional phase caused by the galvanometric scanners, was corrected. This concept has been described by Ginner *et al*. [32]. To reduce the lateral resolution, the resolvable spatial frequencies are lowered. Therefore, the two-dimensional Fourier transformation (2D-FFT) of the whole complex enface OCT data was calculated. There, a 2D Gaussian mask was applied to limit the spatial frequencies. With different size of the Gaussian mask, different lateral resolutions were achieved. Calculating the inverse 2D-FFT, the resolution degraded image was retrieved. The resulting lateral resolution was determined using the ESF method on a resolution target (1951 USAF Resolution Test Target) data set processed with the described method. Previously reported lateral resolutions are within 4-30 µm [20]. The lateral resolution was adapted to 3.7 µm, 6.4 µm, 10.4 µm, 16.6 µm, 25.5 µm, 33.4 µm.

In total, 5 data pairs of corresponding gland and adenoma tissue were adjusted with respect to the axial and lateral resolution. The resolution reduced data was further used for the above described procedure of texture analysis to extract textural features used for computer aided differentiation of pituitary and adenoma tissue in case of compromised resolution performance.

## 3. Experimental results

Out of the collected data, 2 representatives for the embedded and freshly unfrozen biopsies are presented and discussed in detail, respectively.

### 3.1 Identification of morphological features and relevant biomarkers

**Fig. 3.**
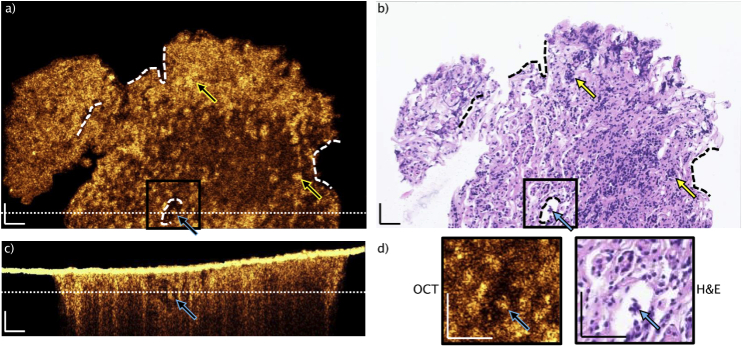
View of typical paraffin block single slice OCT and H&E correlation of pituitary gland without tumor. OCT scan was selected after agreement on matching of structural features (dashed lines: landmarks). Brighter connected structures are visible in the OCT, giving evidence that cell nests (higher cell density and collagen fibers) giving higher OCT signals, when correlated to H&E slice (b). The yellow arrows point to identified nests. The blue arrows are aiming at the same cell group, which can be identified in all displayed images: single OCT enface image (a), H&E slice (b), B-scan (c). The positions of the enface and the B-scan are indicated by the dotted lines, respectively. d) Magnification of the indicated area, showing an isolated cell group. The intense OCT signal in (c) is arising from the air paraffin interface. Scale bars: 50 µm.

Firstly, embedded pituitary tissue examined with the UHR-SD-OCT system and corresponding H&E slice are shown (Fig. 3). Landmarks are identified to enable colocalization in between single scan OCT data and H&E slices: dashed lines are displaying those landmarks (Figs. 3(a) and 3(b)). Thickness of the H&E slice is ∼2 µm and the OCT single scan covers approximately 1.5 µm in depth inside the tissue. The H&E slice presents nests, found in pituitary gland tissue without tumor (yellow arrows in Fig. 3(b)). The pinpointed nests are correlated to the OCT image in Fig. 3(a), generating bright signal. Furthermore, an isolated nest is found in Fig. 3(a), which is indicated by a blue arrow. This is moreover confirmed by the H&E image (blue arrow, Fig. 3(b)). Since the OCT provides depth information, it is possible to identify the isolated nest in the cross-sectional B-scan and its elongation in depth over approximately 50 µm (blue arrow, Fig. 3(c)).

The B-scan position is indicated by the white dotted line in the enface image, Fig. 3(a). The enface image position is indicated in Fig. 3(c) with a white dotted line. Additionally, zoom ins are visualized in Fig. 3(d), in which the isolated nest is pinpointed with a blue arrow. This nest and the identified structures, indicated by the yellow arrows, are giving evidence, that nests give bright OCT signal as a denser structure within the pituitary gland tissue confined by collagen fibers. The increased scattering of the denser cell nests is rather caused by the nuclei to cytoplasm boundary than the nuclei itself as presented before [33,34]. Moreover, the optical density of the nests is increased due to the above-mentioned procedure of fixation and embedding.

**Fig. 4.**
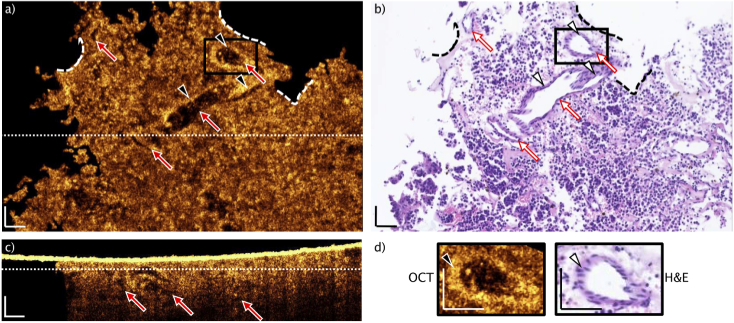
Representative of typical paraffin block single slice OCT and H&E correlation of adenoma tissue. After matching the structural features to the H&E slice (dashed lines: landmarks). No larger connected bright structures, besides the endothelial cells confining blood vessels (triangles) can be identified (d). The red arrows point to blood vessels, indicated in the single OCT enface image (a), the H&E slice (b) and the B-scan (c). The positions of the enface and the B-scan are indicated by the dotted lines, respectively. The intense OCT signal in (c) is arising from the air paraffin interface. Scale bars: 50 µm.

Following, a representative of an embedded adenoma sample is displayed. The same above-mentioned procedure for identifying the correlating position and indicating the landmarks is applied (dashed lines, Figs. 4(a) and 4(b)). Here, the H&E image unveils unconfined cell formations without a reticulin meshwork, but with identifiable vasculature (Fig. 4(b)). Four red arrows in Fig. 4(b) are pointing to vessels, which are correlated to the OCT enface image in Fig. 4(a). The OCT B-scan in Fig. 4(c) provides depth information and exposes additional vessels (red arrows) in a depth of ∼50-100 µm. The white dotted lines in Figs. 4(a) and 4(c) indicate the enface and the B-scan position respectively. If comparing the OCT enface and the H&E image in Figs. 4(a) and 4(b), the only visible larger bright structures are the endothelial cells, lining the inner lumen of the blood vessels (triangles in Fig. 4). One representative blood vessel is magnified in Fig. 4(d), showing a correlation of the OCT and the H&E zoom in, emphasizing the bright appearance of the endothelial structure. Since a long time, the increased vasculature is a well-known biomarker in tumor progression [35–37]. There are also bright, localized intense spots visible in the OCT single enface scan. In comparison to Fig. 3, their size is smaller (<10 µm) and they are scattered over the whole enface scan. It suggests the suspicion that these are individual cells lying loose in the tissue. Essential for understanding and verification of the identified structures and biomarkers – collagen fibers confining cell nests in the gland tissue versus homogeneity in adenoma tissue – is the investigation and translation to fresh biopsies.

### 3.2 Translation to fresh biopsies

**Fig. 5.**
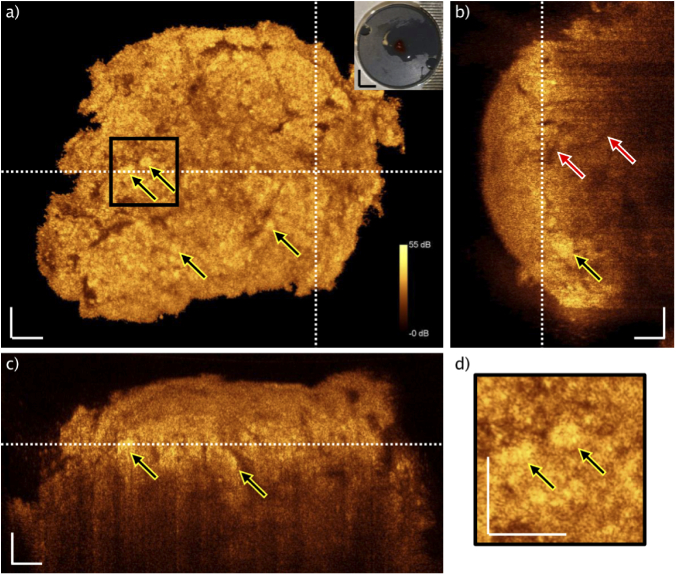
OCT maximum intensity projection of freshly unfrozen biopsy. Color bar is applicable to all images. a) OCT enface image of healthy pituitary gland tissue, nests (yellow arrows). Two white dotted lines indicating positions of the B-scans. Photo inset: mounted biopsy. b) Vertical B-scan with vessel in depth (red arrows) and nest in depth (yellow arrow). White dotted line indicates position of the enface OCT image. c) Horizontal B-scan with two indicated nests (yellow arrows). White dotted line indicates position of the enface OCT image. d) Magnification of the black box in a. Yellow arrows pinpointing the identified nests. White scale bars: 50 µm. Black scale bars: 3 mm.

**Fig. 6.**
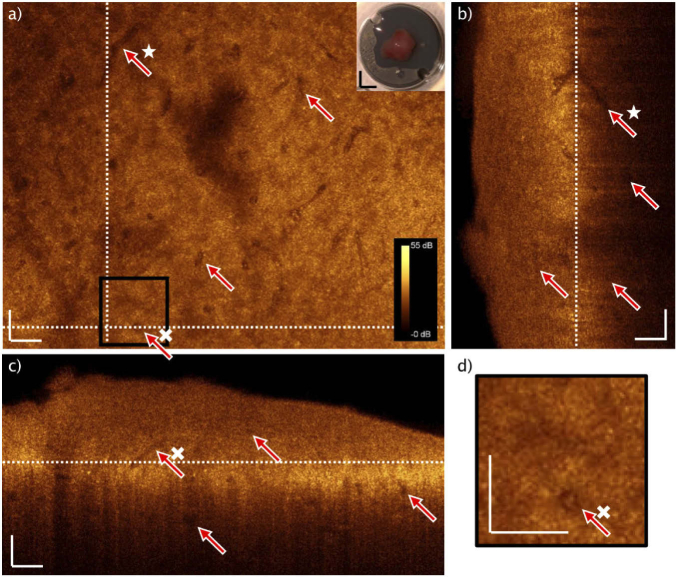
OCT maximum intensity projection of freshly unfrozen biopsy. a) OCT enface image of adenoma tissue, vessels (red arrows). Two white dotted lines indicating positions of the B-scans. Photo inset: mounted biopsy. The star is highlighting a vessel, which is found in depth in (b). b) Vertical B-scan with vessels in depth (red arrows) and a correlated vessel in depth (star). White dotted line indicates position of the enface OCT image. c) Horizontal B-scan with four indicated vessels (red arrows). White dotted line indicates position of the enface OCT image. d) Magnification of the black box in a. Red arrow pinpoints a vessel. White scale bars: 50 µm. Black scale bars: 3 mm.

The identified biomarkers are verified on freshly thawed biopsies: the size of the nests is varying within gland tissue but clearly visible in comparison to the homogeneous appearance in the adenoma case. Especially the presence of the reticulin support structures, consisting of collagen, confining the cell nests giving intense OCT signal. If structures are identifiable in the adenoma tissue, they are most likely blood vessels. In Figs. 5 and 6 OCT data of one representative for gland and adenoma tissue is presented, respectively. Both data sets are presented as maximum intensity projections of 5 subsequent images. In Fig. 5(a), an enface OCT image of pituitary gland is shown. The yellow arrows are pointing to areas with brighter appearance. Out of the previous findings, those are correlating to nests, present in pituitary gland tissue. Since the packing density of those features is higher, the scattering is increased particularly due to present collagen framework, resulting in stronger OCT signal. The photo inset is showing the mounted unfrozen biopsy. Two white dotted lines are indicating the positions of the OCT B-scans (Figs. 5(b) and 5(c)). The large penetration depth and provided depth information of OCT is presented in the B-scans. The vertical B-scan, Fig. 5(b), for instance exhibits a penetration depth up to ∼320 µm. A nest is identified in a depth of ∼80 µm, indicated by the yellow arrow. Furthermore, two red arrows expose one large vessel, which is visible up to a depth of ∼200 µm. The horizontal B-scan (Fig. 5(c)) presents structure and heterogeneity in depth, caused by the nests. Two representatives are pointed by yellow arrows. A magnification of the black box drawn in Fig. 5(a) is presented in Fig. 5(d). This close up highlights two nests.

A representative of freshly thawed adenoma tissue is presented in Fig. 6. In Fig. 6(a), the OCT enface maximum intensity projection of 5 consecutive images is displayed. The red arrows are pointing at vessels, pervading the tumorous tissue in this case. There are many more, but 4 representative vessels are chosen as examples. The white star is highlighting a vessel, which is correlated to Fig. 6(b). The white cross is accentuating one representative, identified in Figs. 6(c) and 6(d). In general, no nest-like structure is visible in the adenoma, which is a biomarker in histopathological examination. The nests are not hold by collagen fibers. The overall appearance of adenoma tissue is more homogenous, because the reticulin framework is lost.

The photo inset shows the mounted biopsy on the microscope holder. The white dotted lines indicate the position of the vertical and horizontal B-scan in Figs. 6(b) and 6(c). The vertical B-scan (Fig. 6(b)) displays information up to a depth of ∼330 µm and the enface (Fig. 6(a)) position is at the white dotted line. Similarly, vessels are visualizable in depth of ∼250 µm. Additionally, more shallow located vessels in depth of ∼120 µm can be observed. The vessel, tagged with the white star, is correlated to the vessel in Fig. 6(a). It shows an intersecting blood vessel in depth. The horizontal, cross-sectional B-scan in Fig. 6(c) indicates the enface maximum intensity projection (Fig. 6(a)) position with the white dotted line. Here, vessels up to a depth of ∼230 µm can be identified. Again, the overall adenoma appearance is more homogenous in the OCT. The vessel, emphasized by the white cross, is partly lengthwise imaged here but cut transversely in the enface (Fig. 6(a)).

This vessel is further magnified in Fig. 6(d), indicated by the black box in Fig. 6(a). The enface image of the transversely cut vessel might be confused with an isolated nest, as found in Fig. 3. Since OCT provides the advantageous cross-section in depth, the horizontal B-scan (Fig. 6(c)) unveils the vascular appearance of this feature.

**Fig. 7.**
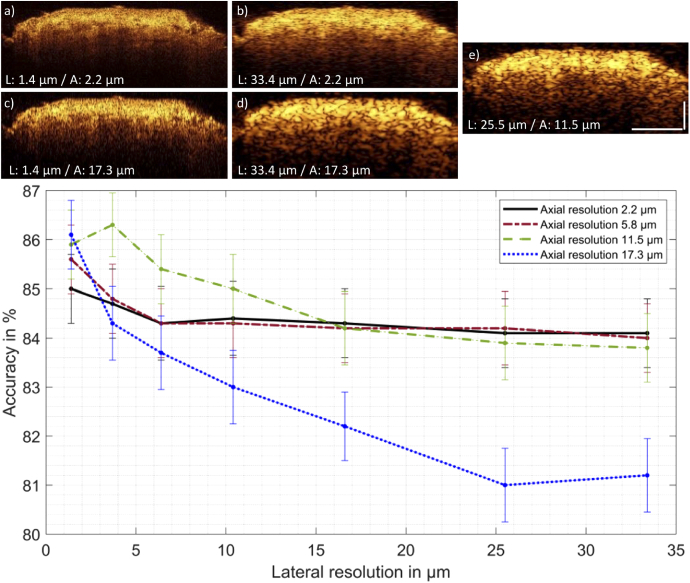
Classification of OCT data of pituitary and adenoma. Top: Five representative OCT B-scans of pituitary gland for the extreme cases of lateral (L) and axial (A) resolution adjustments are shown a)-d). e) provide comparable resolution realized in an endoscope applied before [31]. The pituitary adenoma images are treated in the same way and express the same behavior after optical resolution adjustments. All five B-scans have the same dynamic range. Scale bars: 200 µm. Bottom: Achieved accuracy of the classification performed on the chosen OCT data. Error bars represent the 95% confidence interval. The lines in between the data points are no interpolations but serve visual purposes. The qualitative interpretation of the classification outcome indicates, that with decreasing axial resolution the decreasing lateral resolutions has the strongest impact on the classifier’s accuracy. The accuracy is higher than 80% for all cases.

In comparison of Figs. 5 and 6, the photo insets show similar textural features for the tissue itself, resulting in difficulties of visual differentiation. However, OCT provides the capability of analyzing structural features in depth to gain more information of identified structural features. The main morphological difference is the homo- and heterogeneity of the tissue regarding well defined nests (heterogenous) in the healthy case (Fig. 5) and the disorganized adenoma case (Fig. 6) where nests vanish (homogenous). Additionally, the present reticulin/collagen fiber framework in gland tissue is causing higher OCT signal, emphasizing the heterogenous appearance with the cell nests. Since Figs. 5 and 6 have the same dynamic range, the brightness comparison becomes an obvious differentiation parameter. In the following, the defined biomarkers are quantified.

### 3.3 Image analysis and evaluation of reduced optical resolution for potential in vivo endoscopic use

To investigate and estimate endoscopic OCT performance for differentiation of adenoma and pituitary tissue, data sets with adjusted lateral and axial resolution are analyzed with the explained textural feature extraction and classification algorithm to find the optimal trade-off between optical performance and applicability in a clinical *in vivo* setting. The PCA allows for the reduction of the original data dimensionality via an orthogonal transformation and provides a representation of the input data in a lower-dimensional space through the calculation of a new orthogonal basis vector [30]. PCA was used to obtain information about the highest variance on the selected features. Although PCA is suitable for dimensional reduction and feature interpretation it only gives a small insight on performance, hence a support vector machine algorithm (SVM) was employed to classify and differentiate the OCT data between adenoma and gland tissue. Leave-one out cross-validation (LOOCV) was selected as the evaluation method for the collected data. In this special case of cross-validation, the number of folds is equal to the number of sample pairs. Therefore, a total of 5 SVMs were trained using an RBF (radial basis function) kernel (C = 0.01 and automatic gamma selection). For each SVM four pairs of biopsies were used for training and the last remaining pair was used for testing. Each pair of biopsies contained a total of 1000 B-scans from which 2000 patches were extracted and used for the evaluation. Due to the low diversity of samples represented by our dataset this was the preferred method for this study.

All five shown B-scans in Fig. 7 have the same size of 1600 × 615 pixels (lateral x axial) and the same dynamic range. They show the boundaries for the lateral and axial resolution reduced images. Modifying the resolution of the data leads to increased speckle size, especially towards resolution values of Fig. 7(d). It appears, that with decreasing axial and lateral resolutions, the signal contrast at larger penetration depth increases due to enlarged speckles in greater depth.

The first principle component (PC1) of the original data pair (compare Fig. 7(a)), namely energy, explains 80.2% of the variances for the classification. For all analyzed levels of resolution, the PC1, indicating the highest variance between the two data sets, is either correlation or energy of the GLCM. For instance, the PC1 indicates 67.7% of the variances for the correlation feature between adenoma and gland data for the case shown in Fig. 7(b), with a lateral and axial resolution of 33.4 µm and 2.2 µm, respectively. Depending on which of the two textural features is more pronounced within one pair it serves as the first principle component for the classification. This is well in line with the observations in the OCT data of fresh adenoma and pituitary biopsies (representatives shown in Figs. 5 and 6): correlation of pixels in the heterogeneous appearance of the pituitary gland and its cell conglomerations and energy indicating the homogenous structure of an image, applicable for the adenoma data.

The classification’s accuracy result in case of modified resolution for pituitary/adenoma tissue is shown in Fig. 7. For all investigated cases of lateral and axial resolution, the accuracy of introduced classification is higher than 80%. The error bars indicate the 95% confidence interval. For mostly all axial resolution settings, the confidence intervals are overlapping in the range from 1.4-10 µm lateral resolution. The trends for different axial resolution in dependency of the lateral resolution indicates, that with decreasing axial resolution the decreasing lateral resolution has the most recognizable adverse impact on the classification accuracy.

## 4. Discussion

Our findings reveal the ability of UHR-OCT to provide information for an onsite pituitary gland and adenoma differentiation, which is so far only accessible after histopathological processing and analysis. Here, we focused on morphological biomarker identification in OCT images. The use of OCT on this tissue type has never been reported before. The 800 nm UHR-SD-OCT system enables high contrast and high isotropic resolution (sub 2.5 µm) OCT images on pituitary gland and adenoma tissue. Moreover, the acquired images give insight on using OCT at high optical performance (contrast, resolution and penetration depth) even in paraffine embedded tissue. They can be compared and correlated to histopathological images, defining expectable outcome and visualized structural features applying OCT.

Muller *et al*. presented a method in order to achieve one-to-one co-registration of OCT images to H&E slices [14,38]. For the purpose of achieving immediate differentiation of gland and adenoma tissue, preferably *in vivo*, we performed direct UHR-SD-OCT imaging on embedded biopsies and correlated the results to H&E images to define morphological biomarkers (Fig. 3 and Fig. 4). Limitations, permitting the one-to-one co-registration in our case, are arising from the processing of embedded biopsies. OCT imaging and the H&E slicing are not ensured to take place in the same plane, since the paraffin block could be mounted slightly angled in the slicing machine and the present focus plane curvature prevents high lateral resolution in a flat plane. The density of paraffin embedded tissue causes further limitations for OCT imaging: the air-paraffin interface is highly reflective and index matching is difficult. The hydrophobic characteristic of paraffin prevent water as an index matching medium and more viscose liquids like ultrasonic gel cause structural features interfering with the real structures from the embedded tissue. Moreover, the high density of paraffin is causing even higher scattering at 800 nm and causing increased background signal. However, it is possible to retrieve important insight to biomarkers from the embedded tissue imaging.

The histopathological biomarker out of H&E pituitary gland images are the cell conglomerations, known as nests [7], confined by the reticulin fiber framework. Using the advantage of volumetric OCT and its possible enface projection for correlation to H&E, we demonstrate in Fig. 3 that OCT imaging provides information on this important biomarker. Contrarily, adenoma tissue is characterized by the loss of confining collagen fiber and broken apart nests [9]. In Fig. 4, no bright structures are visible in the OCT, except the cohesive, endothelial structures (Fig. 4(d)). The homogenous appearance in comparison to Fig. 3 is dominant, confirming the ability of OCT to target the histopathological biomarkers for gland and adenoma differentiation. Additionally, blood vessels are identified in the OCT correlation of embedded adenoma tissue to H&E images.

The known morphological biomarker for pituitary gland and adenoma (presence vs. absent nests) is confirmed in the UHR-OCT data taken on freshly unfrozen biopsies (Figs. 5 and 6). There, the enface OCT shows a heterogeneous appearance due to the present nests (Fig. 5(a)). The increased scattering is leading to brighter structures and causing the heterogeneity. Contrarily, the adenoma is showing uniformity. The structured morphology is broken and the OCT enface of the adenoma is appearing more homogenous (Fig. 6(a)). The blood vessel confining endothelial cells are not visible in the unfrozen biopsies (Fig. 6), which might be due to the increased density after the fixation and embedding procedure [39,40]. For a definite comparison of the found features, a transition zone of adenoma and pituitary tissue would be favorable.

In comparison to common imaging tools like fluorescence microscopy, confocal microscopy, or ultrasound, OCT provides enhanced depth information and penetration with quasi isotropic high resolution. For instance, the conventional fluorescence techniques are only able to detect adenoma tissue near the surface and provide specificity below 20% [41]. Also, the similar uptake of fluorescence agents in pituitary or adenoma tissue is additionally cumbersome [42]. Contrariwise, OCT provides high imaging speed, volumetric three-dimensional information in depth over a large FOV, which is implementable in endoscopic devices.

Furthermore, an approach for estimation of classification performances based on optical resolutions realized in endoscopic probes is presented. Out of the UHR-SD-OCT images, axially and laterally resolution reduced OCT data sets are generated. Representative B-scans are shown in Fig. 7. For a localized differentiation between healthy and diseased tissue it is generally preferred to have small patch sizes for performing software-based classification. The reason why 200 × 200 pixel sized patches are taken into the texture analysis becomes obvious looking at the speckle size in Fig. 7(d). With increasing speckle size, a smaller patch would only include same valued pixels, resulting in a homogenous patch and by that jeopardizing the texture analysis. The classification outcome, namely accuracy in Fig. 7 gives qualitative insight to the classification performance. The textural feature analysis with subsequent principle component analysis indicate the energy and correlation as PC1 in all cases. This gives quantitative proof of the visually identified structural features serving as biomarkers for clinical differentiation of adenoma and gland tissue. The trend of the data shown in Fig. 7 is indicating, that with reduced axial resolution the decreasing lateral resolution is having the highest impact on the accuracy.

Towards *in vivo* endoscopic applications, the axial resolution, dependent on the used laser system with a certain bandwidth and central wavelength, could potentially compensate for intrinsic lower lateral resolution of the endoscopic probe. Surely, there is also a trade-off between high lateral resolution and the imaging depth of OCT. To detect invasively grown adenoma a high depth range is favorable, which is limiting the lateral resolution on the other hand. Referring to Fig. 7, less than 10 µm lateral resolution would enter the range of optical coherence microscopy (OCM) with its high lateral resolution reducing the imaging depth range. Since the used data set is small, especially the high resolution region lower than 10 µm lateral resolution with the overlapping confidence intervals needs careful interpretation. In this region with high amount of resolved textural features, the used data set is not giving a robust classifier outcome. Obviously, a much larger data set would help the algorithm to deliver more stable results. Moreover, the resolution reduction is performed on the same root image acquired with the UHR-SD-OCT microscope, leading to a strong bias of the created data. Additionally, the classification performance is affected by the pathological label per biopsy. The classifier is treating single B-scans of one biopsy data set individually being sensitive to transitions of pituitary/adenoma, while the pathological label is treating one biopsy as a whole. Training the classification algorithm with false labeling causes reduced performance. Nevertheless, all retrieved accuracy levels are above 80% giving strong evidence for the relevance of OCT in this field. In particular, the 12 µm axial and 28 µm lateral resolution region which can be achieved with an endoscopic probe developed by the authors previously [31] achieves an accuracy well above 80%. This probe is working at 1300 nm central wavelength resulting in lower contrast but with higher penetration depth, being beneficial for the screening of invasive tumor fragments. This parameter needs further investigation in forthcoming studies, e.g. more data sets of pituitary and adenoma tissue acquired with different wavelengths would unveil a detailed insight to resolution and contrast dependent classification abilities. In this case, the separation of the investigated resolution values (lateral and axial) with respect to the accuracies achieved in this study (see Fig. 7), are expected to become more pronounced.

During the standard procedure of pituitary adenoma resection, rigid endoscopes are introduced to the sella turcica, the location of the pituitary gland. Since the nose provides two openings for introducing instruments, one would be used for standard white light visualization tools and an OCT probe could be introduced through the other. Correct positioning of the OCT probe could be guaranteed with the white light visualization of the used standard instruments. Therefore, no further restrictions on rigid lengths of the OCT probe are given as boundaries from the used equipment in neurosurgical departments. The provided working channel diameter of such rigid tubes for entering the location of the pituitary gland are in the range of 6-7 mm.

Endoscopic probes incorporating OCT have been applied for in-vivo applications in many medical fields for instance in esophagus [43,44], colon [45,46] or intravascular applications [47,48], proofing the translation of OCT into miniaturized imaging devices. In addition, endoscopic angiography has been demonstrated recently, providing possible additional contrast for the pituitary gland and adenoma differentiation [49]. Using 800 nm for in-vivo endoscopic application would lead to significant light absorption, due to the hemoglobin characteristics and high number of scattering red blood cells during surgery [50,51]. Nevertheless, flushing of the inspected area during pituitary adenoma resection would be required anyway for orientation purposes of the neurosurgeon. In further investigations, the overall minimal requirements could be examined in detail so that more precise statements can be made about the trends found here. In a subsequent study, the robustness of the identified criteria could be explored by sophisticated computer aided analysis like neuronal networks including a significant higher number of samples. The method introduced here can be used to evaluate development parameters for probe development in advance and thus develop more targeted and resource-efficient endoscopes.

In conclusion, OCT has shown to be a suitable technique for differentiating pituitary gland and adenoma. Up to now there is no distinct technique available that clearly differentiate between gland and adenoma tissue sufficiently onsite during neurosurgical intervention. The superficial tissue discrimination during surgery is nowadays based on visual inspection and the experience of the neurosurgeon. Maintaining the regulation of endocrinological processes by the total adenoma resection and the preservation of the pituitary gland is critical for the wellbeing of a patient. Therefore, a quantifiable measure is required to increase successful adenoma resection and reduce hormone treatment in case of lost gland function. We demonstrated that this is possible with OCT. In comparison to the used techniques such as fluorescence guidance or microscopy nowadays, OCT could furthermore be used for detecting invasive tumor fragments in real time and three dimensions because of the retrieved depth information and provide a clear onsite differentiation capability to neurosurgeons with accuracies higher than 80%.
